# Associations of multiple evidence-based care strategies with disease mortality, life expectancy, and disparities in the United States

**DOI:** 10.1093/haschl/qxag184

**Published:** 2026-07-28

**Authors:** Douglas A Corley, Mubarika Alavi, Bruce H Fireman, Tori Finch, Stephanie Prausnitz, Smita Rouillard, Kristine Lee, Lawrence Hamilton, Maria Ansari

**Affiliations:** Kaiser Permanente Northern California, The Permanente Medical Group, Pleasanton, CA 94588, United States; Kaiser Permanente Northern California, The Permanente Medical Group, Pleasanton, CA 94588, United States; Kaiser Permanente Northern California, The Permanente Medical Group, Pleasanton, CA 94588, United States; Mid-Atlantic Permanente Research Institute, Washington, DC 20002, United States; Kaiser Permanente Northern California, The Permanente Medical Group, Pleasanton, CA 94588, United States; Kaiser Permanente Northern California, The Permanente Medical Group, Pleasanton, CA 94588, United States; Kaiser Permanente Northern California, The Permanente Medical Group, Pleasanton, CA 94588, United States; Kaiser Permanente Northern California, The Permanente Medical Group, Pleasanton, CA 94588, United States; Kaiser Permanente Northern California, The Permanente Medical Group, Pleasanton, CA 94588, United States

**Keywords:** chronic disease mortality, integrated care, delivery science, disparities

## Abstract

**Introduction:**

Limited data exist regarding the potential cumulative effects of multiple, integrated, system-level strategies on population-level outcomes and disparities.

**Methods:**

We evaluated associations between membership within a multi-disease, integrated population health management model with disease-specific mortality, life expectancy, and demographic disparities and relevant comparators. Bias was minimized through use of a large, representative, multicenter community-based setting, multiple comparators (including similar insured status, same-state, comparable comorbidity distributions, and national), demographic adjustments, and causality criteria.

**Results:**

Among 3 944 173 persons demographics-adjusted mortality was significantly lower for 9 of 10 leading causes of death, including the 5 leading preventable conditions (age-, sex-, race-adjusted risk ratios and 95% CIs Kaiser Permanente, Northern California vs United States [US, all])—cancer (0.85; 0.83-0.88), heart disease (0.69; 0.67-0.71), stroke (0.86; 0.82, 0.91), injury (majority overdoses and falls; 0.45; 0.42-0.48), and respiratory disease (0.59; 0.55-0.63)—but not unmodifiable causes (eg, Alzheimer's disease (1.58; 1.51-1.66). Most life-years gained occurred during ages most impacted by disease-management programs: 50–72 years (>0.075 life-year/year). Life expectancy exceeded comparators and was 4.9 years higher than the US average (83.3 vs 78.4 years, 2023). Findings were robust to index years, multiple other comparator populations, and adjusted analyses. Demographic disparities were smaller across major benchmarks.

**Conclusion:**

These findings suggest that high uptake of multiple, existing, evidence-based population health strategies may be cumulatively associated with substantially reduced disease-specific mortality, decreased disparities, and increased life expectancy, comparable to values in comparator countries.

## Introduction

Improving the length and quality of life are fundamental health care goals, yet few studies exist regarding the potential net impacts of multiple, high-uptake, evidence-based population care strategies on decreasing deaths from chronic diseases and their potential cumulative influences on population-level life expectancy. Observed variations indicate substantial opportunities for decreasing premature causes of death. In 2023, for example, life expectancy was 84.7 years in Japan, 83.7 in Spain, 83.9 in Australia, and 79.3 in the United States.^1^ Within the United States alone, it ranges between states by 9 years, from 79.9 to 70.9.^2^

Among the 5 leading causes of death in the United States, approximately 40% might be prevented or delayed through modifiable risk factors.^3^ However, despite substantially increased health care expenditures and access, avoidable mortality has been increasing in the United States while decreasing in other countries.^4^ Identifying effective strategies for delivering existing evidence-based care across multiple disease entities is a feasible path for reversing these trends and creating higher-value health care. Health care settings with multiple evidence-based health care interventions are a theoretical test setting for estimating longer-term effects on health care outcomes and life expectancy across large populations.

In a population with high uptake of preventive services and disease-management programs, the current study examined associations with life expectancy, disease-specific mortality, and projected life-years gained in comparison to relevant national and statewide comparators, including by sex, race, and ethnicity. The study used several strategies to address potential confounding factors, including use of a large, community-based setting with long membership durations and comparable confounder distributions to its underlying regional census population; adjustment of patient demographics between comparators; multiple comparators (including low-uninsured population, same-state population, and comparable morbidity profile populations); variations across causes of death with variable modifiability; and assessment by conventional causality criteria.

## Data and methods

### Study populations

The study evaluated a multicenter, integrated health care delivery system (Kaiser Permanente Northern California [KPNC]). Members are comparable to the underlying region's census population by age, race, ethnicity, sex, educational achievement, household income, and a social vulnerability index.^5^ Thus, studies within this setting closely approximate population-based studies by available metrics.^5^ It provides care across 21 geographically dispersed medical centers, has approximately 9500 physicians, includes substantial variability by payor type (Medicare, Medicaid, and commercial insurance), and has high retention rates (see Results), providing the ability to evaluate multi-year exposures. The 2017–2023 study population included approximately 4 million members, which comprise more than 30% of the region's underlying population; only 5.1% of the regional population is uninsured.^5^

The study setting uses multiple evidence-based population screening and care approaches for cancer screening, blood pressure control, cardiovascular disease, diabetes, opioid overdose prevention, fall risk, depression, etc.^6-12^ These programs have high uptake, as measured by National Committee for Quality Assurance ratings for successful implementation (Table S1).^13^ Implementation approaches include outreach to people not actively seeking care. Individual program effectiveness evaluations have included uptake and disease outcomes against internal and external comparators.^6-12^

Comparators, selected blinded to mortality and life expectancy data, included populations comparable to or adjusted for major confounders. These included California (all, which includes KPNC), a southwestern state (Arizona), a western state (Washington) with comparable comorbidity profiles (see later in text), and the United States (all). We also reference the highest (Hawaii) and lowest (Mississippi) life expectancy states. Given that chronic disease prevalence and social factors may influence life expectancy, state-level comparators had similar Centers for Disease Control and Prevention (CDC) hot-spot mapping for 10 common chronic diseases.^14^ The mapping also closely approximates state-level distributions of obesity and Healthy People 2030 Social Determinants of Health domains, including economic status, Supplemental Nutrition Assistance Program status, race, ethnicity, insurance, and social disadvantage proxies.^14,15^

### Data sources

Mortality data used California death certificates, the Social Security Death Master File, and medical coding. Life expectancy calculations used the most recent data from the US CDC’s National Center for Health Statistics for the United States (2017–2023) and stratified by state (2017–2021)^2^ and KPNC membership data.

### Outcomes

Primary outcomes included disease-specific mortality for the top 10 causes of death, age-specific life-year contributions, and total life expectancy from birth. The 5 leading preventable causes followed CDC definitions: heart disease, cancer, unintentional injury, chronic respiratory disease, and stroke.^16^ The most common unintentional causes of death among people younger than 45 years is poisoning (mainly drug overdoses, >60%)^17^ and among people 65 years and older is falls (>50%).^18^ Additional analyses examined disparities for available comparators by sex, race, and ethnicity. Race and ethnicity were defined using the electronic medical record, which incorporates self-report, registries, and other data sources.

### Statistical analysis

Age- and cause-specific mortality rates used the most recent data from the CDC and, for direct standardization, US age, sex, and race distributions (2023).^2^ Life expectancy estimates used life tables and annual age-specific year-specific mortality (2017–2023), consistent with National Center for Health Statistics' methodologies.^19^ These calculate mortality rates across 101 age groups, from the 1st to the 100th year of life, plus persons aged 100 years and older. The 2023 mortality rate for 72-year-olds, for example, would use deaths during 2023 at age 72 divided by the total persons aged 72 years old midyear. This creates a hypothetical cohort of 100 000 newborns, subject to the age-specific mortality rates for 2023. For each year lived, the table provides deaths, survivors, and survivors' average remaining years of life—that is, their life expectancy. A life expectancy estimate predicts how long infants born in that year would live if they experienced the age-specific death rates prevailing for that index year. The KPNC estimates were compared with reported national and state-specific life expectancy estimates from the CDC.

Age-group level contributions to overall life expectancy differences used disaggregated life expectancies.^20^ The person-year contributions of mortality differences in the 70th year of life to the total life expectancy difference from birth, for example, would be driven by the US minus KPNC mortality difference at age 70 and the remaining years of life at age 70.

The estimated 95% CIs for the KPNC vs US comparisons used 1000 parametric bootstrap simulations for each subpopulation by age, race, and ethnicity to create a simulated life expectancy table, using random samples from a binomial distribution. Analyses and results are reported consistent with EQUATOR reporting guidelines, specifically the STROBE checklist for observational studies.

## Results

### Population

The study population distributions by sex were generally similar to California (all) and the United States (Table 1, also includes population size). The most recent data are from 2023 for the United States (all) and from 2021 for individual states. While study population demographics closely approximate the underlying Northern California population, KPNC members were slightly older than California (all) and the US populations (median: 41 vs 38.8 and 37.0 years, respectively).^5^ By race and ethnicity, KPNC had a higher proportion of people who were Asian compared with nationwide or statewide; a higher proportion of Hispanic people compared with nationwide, but lower compared with statewide; and a lower proportion of Black people compared with nationwide, but higher compared with statewide (Table 1). Among 2023 members (index year), more than 90% of people aged 65 years and older and more than 81% of people ages 40–64 years had been KPNC members for 5 or more years, with regard to opportunities for long-term care exposures and attrition for outcome ascertainment.

**Table 1.** Demographic characteristics for Kaiser Permanente Northern California and the United States (all), year 2023, and for Kaiser Permanente Northern California, United States (all), and California (all) populations, year 2021.^a^

	2023		2021		
	KPNC (n = 3 944 173)	United States^b^ (n = 336 806 231)	KPNC (n = 3 908 079)	United States^b^ (n = 332 099 760)	California^c^ (n = 39 229 543)

Race/ethnicity, %					
Black	6.5	12.6	6.5	12.6	5.6
Asian	21.3	6.3	20.8	6.0	15.3
Hispanic	23.1	19.6	22.4	19.0	40.2
Other	3.1	3.4	3.2	3.3	3.8
Unknown	6.5	0.0	5.6	0.0	0.0
White	39.5	58.1	41.5	59.2	35.2
Sex					
Female	51.5	50.5	51.6	50.5	50.0
Male	48.4	49.5	48.3	49.5	50.0
Other/unknown	<0.1	NA	<0.1	NA	NA
Age					
Median age, y	41.0	39.0	41.0	38.8	37.0
<20 y, %	21.5	24.4	22.0	24.8	24.8
20-39 y, %	25.6	27.0	26.1	26.9	28.4
40-54 y, %	20.8	18.6	20.7	18.6	19.0
55-64 y, %	13.6	12.5	13.9	12.9	12.1
65-79 y, %	14.4	13.7	13.6	13.1	11.7
80+ y, %	4.1	3.9	3.9	3.7	4.0

Abbreviations: KPNC, Kaiser Permanente Northern California; NA, not available.

^a^The most recent data available for the United States are for 2023. The most recent per-state data available are for 2021; thus, comparisons provide both years.

^b^United States population (all).

^c^California population (all, including KPNC).

### Cause-specific mortality

**Figure 1.**
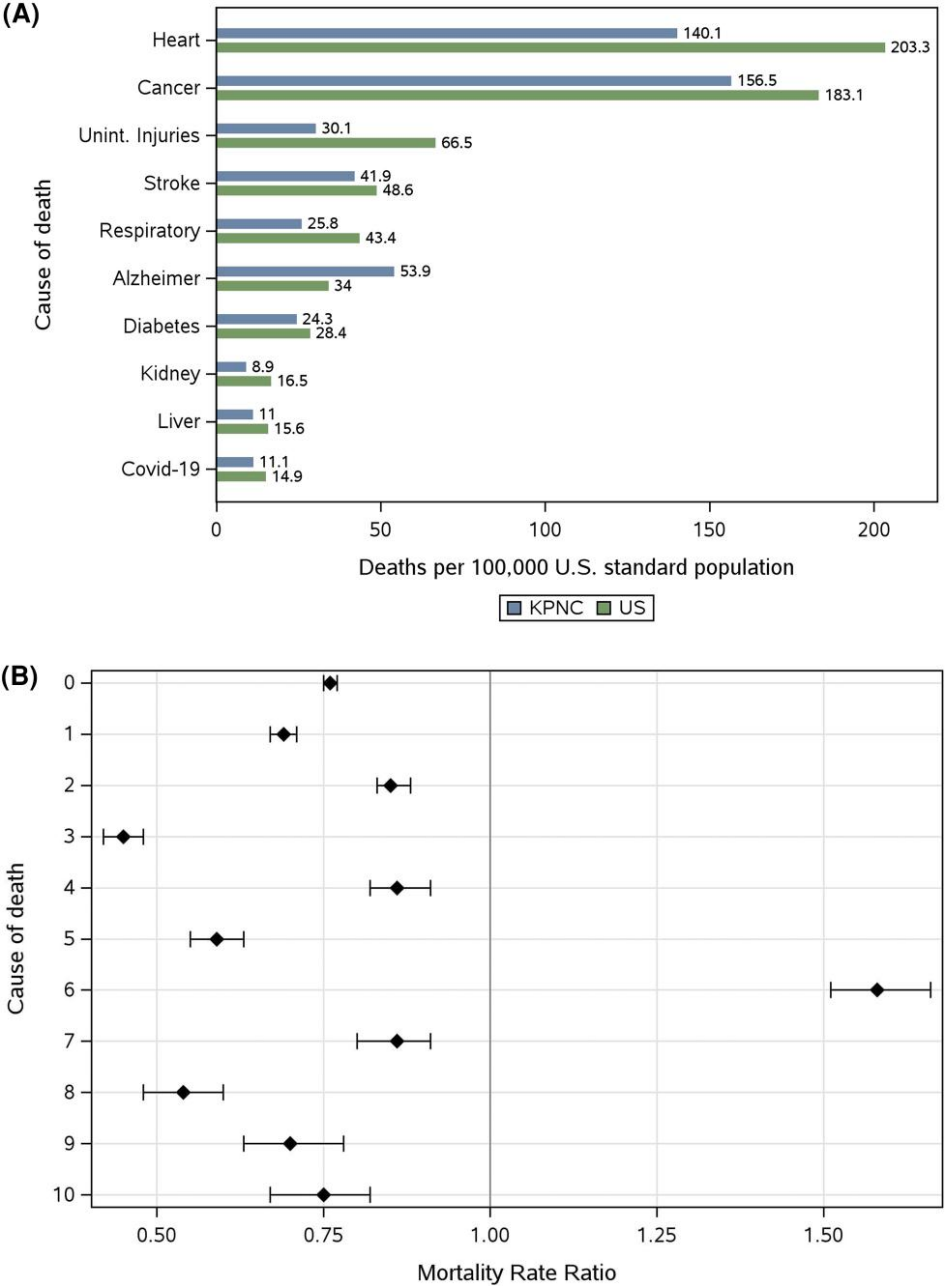
(A) Death rates for top-10 leading causes of death; KPNC vs United States (per 100 000 population, KPNC age-, race-, sex-standardized to the US population, 2023). (B) Rate ratios for top 10 causes of death; KPNC vs United States (KPNC mortality rates age-, race-, sex-standardized to the US population). Values are directly standardized ratios and 95% CIs, 2023. Abbreviations: KPNC, Kaiser Permanente Northern California; Unint., unintentional.

Cause-specific mortality adjusted for patient demographics was lower in the study setting vs United States for 9 of 10 leading causes of death (Figure 1A, year 2023). These included the 5 leading preventable causes (age-, sex-, race-adjusted risk ratios and 95% CIs, Figure 1B)—cancer (0.85; 0.83–0.88), heart disease (0.69; 0.67–0.71), stroke (0.86; 0.82–0.91), unintentional injury (majority drug overdoses and falls) (0.45; 0.42–0.48), and respiratory disease (0.59; 0.55–0.63)—but not for unmodifiable causes (eg, Alzheimer's: 1.58; 1.51–1.66). Evaluation of a similar condition with few strategies for screening and mortality reduction, Parkinson's disease, had similar associations, with 2023 age-, race-, sex-standardized mortality rates of 13.3 and 11.9 per 100 000 for the KPNC and US populations, respectively. Results were robust to data year (eg, 2021 vs 2023), except for a stronger protective association for COVID-19 in 2021 (0.45; 95% CI: 0.43–0.47) than in 2023 (0.75; 95% CI: 0.67–0,82).

For all causes combined, the mortality rate ratio (study population vs US, 2023) was 0.76, (95% CI: 0.75–0.77; age-, sex-, race/ethnicity-standardized). Age-specific mortality was significantly lower in the study setting in every age group and was more than 40% lower than nationwide rates for ages 20–70 years. Mortality ratios attenuated at older ages but remained significant at less than 1.0.

### Age-specific life-year differences

**Figure 2.**
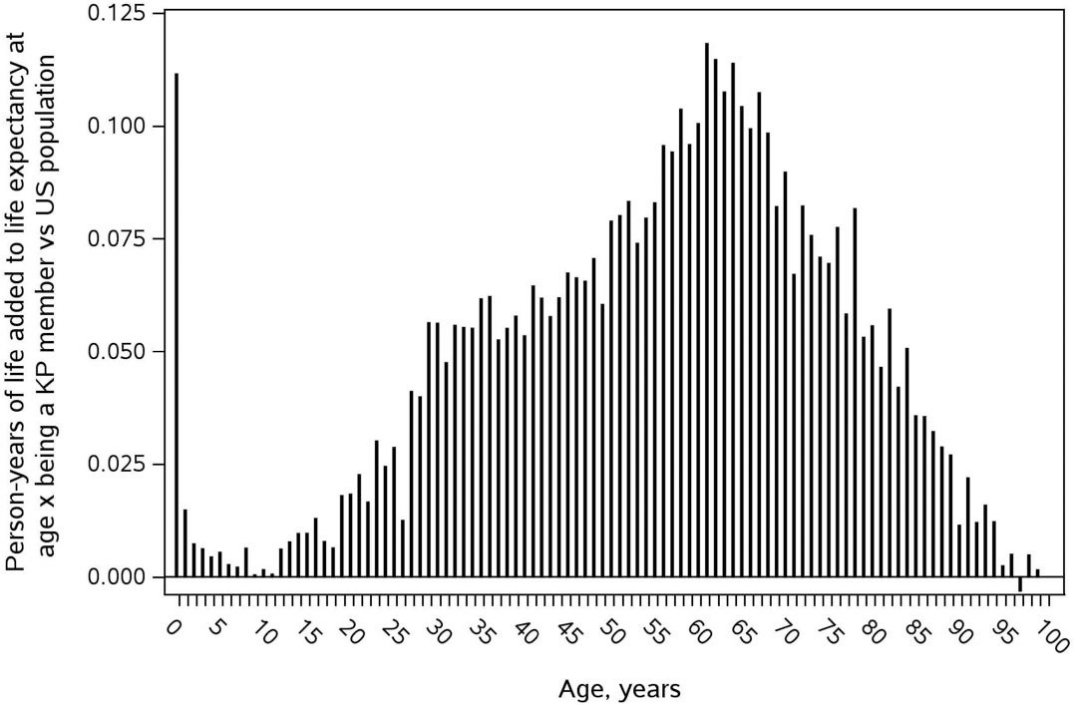
Distribution of additional life expectancy by year of age, KPNC vs United States (person-years, 2023). Abbreviation: KPNC, Kaiser Permanente Northern California.

Between the study population and the United States, additional life-years accrued mainly between ages 29 and 78 (>0.05 additional life-/age-year), with the greatest accruals between ages 50 and 72 (>0.075 life-year/age-year most years; Figure 2). Older age groups contributed progressively fewer life-year differences, given both fewer person-years gained per death delayed and fewer people alive at each age. Deaths among younger age groups were infrequent; thus, although each contributed many life-years, they added few total life-years, except for the first year of life.

### Overall life expectancy

Study population members' projected life expectancy at birth exceeded all comparator populations and across all age groups. Life expectancy from birth exceeded the United States by 4.9 years (83.3 vs 78.4 years; *P* < .01; 2023). Results were robust to sensitivity analyses by index year (2017–2023; data not shown). Results were also robust to multiple comparator state populations (2021; most recent year), including same state, California (which included KPNC, 4 years; 82.3 vs 78.3 years; *P* < .01), highest-longevity state (Hawaii: 2.4 years; 82.3 vs 79.9 years), comparable comorbidity states (Washington: 4 years; 82.3 vs 78.2 years; and Arizona: 7.3 years; 82.3 vs 75.0 years); adjustments for demographics (Table 2); and by causality criteria (see Discussion). Life expectancy differences persisted across different index ages. For example, for the US comparator, the additional projected life expectancies were approximately 3 years from age 50 (35.0 vs 31.8 years) and more than 2 years from age 65 (21.4 vs 19.5 years; index year 2023).

**Table 2.** Life expectancy by sex, race, and ethnicity, Kaiser Permanente Northern California vs United States (2023), for 2 comparators: starting at birth and starting at age 65 years.

	Life expectancy starting at birth, y		Life expectancy starting at 65 y,^a^ y	
	KPNC (95% CI)	US	KPNC (95% CI)	US
Race/ethnicity				
Black	79.7 (79.1-80.3)	74.0	19.8 (19.4-20.1)	18.2
White	82.9 (82.7-83.0)	78.4	21.0 (20.9-21.1)	19.3
Asian	85.9 (85.5-86.2)	85.2	23.6 (23.3-23.8)	23.2
Hispanic	82.7 (82.3-83.0)	81.3	21.1 (20.9-21.4)	21.6
Other (KPNC only)	86.8 (86.1-87.5)	NA	23.4 (22.9, 24.0)	NA
Native Indian/Alaska (US only)	NA	70.1	NA	18.9
Sex				
Female	84.9 (84.7-85.1)	81.1	22.5 (22.4-22.6)	20.7
Male	81.5 (81.3-81.7)	75.8	20.2 (20.0-20.3)	18.2
All	83.3 (83.1-83.4)	78.4	21.4 (21.3-21.5)	19.5

Abbreviations: KPNC, Kaiser Permanente Northern California; NA, not available.

^a^Evaluates life expectancy and projected mortality events beyond age 65 y, for those living to age 65 y.

### Life expectancy by demographic group

Projected life expectancy differences/gains were seen across all demographic subgroups by sex, race, ethnicity, and age (Table 2); the largest gains were among groups with the largest disparities (Table 2; 2023). For example, life expectancy differences were 3.4 vs 5.3 years between men and women in the study population compared with the US populations, respectively (*P* < .01); comparable patterns were found using the all-California population. Similarly, life expectancy differences were 3.2 vs 4.4 years between Black and White people, for the current KPNC study population compared with the overall US population, respectively (*P* < .01) (Table 2).

### Temporal trends

Life expectancy projections and differences were robust to index year for both US (2017–2023) and most recent state (2017–2021) data. Annual changes in life expectancy had similar patterns, although the magnitude of life expectancy decreases during the pandemic years of 2020 and 2021 was lower in the study than in comparator populations. Both population's life expectancies returned to close to pre-pandemic levels by 2023.

## Discussion

Evaluation of a large, multicenter, representative population using multiple high-uptake, evidence-based chronic disease–management strategies found associations with significantly lower mortality for modifiable conditions, reduced demographic disparities, and increased life expectancy relative to diverse comparator groups, including populations with similar comorbidities, low uninsured rates, and after adjusting for age, sex, race, and ethnicity. The findings support and extend results from prior single-disease trials and population studies^6-12,21-23^ to estimate the potential cumulative potential mortality impact of multiple coordinated interventions within a large, multicenter setting.^24^ The results also extend prior studies by providing, beyond total life expectancy, disease-specific mortality, distributions of life-years gained by age, detailed analyses for disparities, and post-pandemic data.^25^ There are few similar comparators we could identify in other settings, as most either evaluate life expectancy for a single disease or for national health systems without direct relevant comparators.^26,27^ For example, a same-state colorectal cancer study found that people within similar systems were more likely to receive guideline-recommended treatments, had improved survival, and had decreased disparities than comparison populations, even after detailed propensity matching for multiple potential confounders.^28^ Similarly, detailed within-population studies exist, which decreases the potential for bias. For example, heart disease and stroke mortality within the current study's setting declined substantially faster than national trends after implementation of a hypertension management program that doubled hypertension control.^9,29^ Similarly, an integrated regional telestroke program markedly decreased time to thrombolysis for acute stroke^12^ and a population-level colorectal cancer screening intervention was associated with a within-population doubling of screening rates and a subsequent 50% decrease in cancer-related mortality, including markedly decreased within-population demographic disparities.^30^

The current study has several potential limitations. First, although the study setting uses evidence-based interventions that are likely effective, the population-level effect estimates herein were not adjusted for individual-level potential confounding factors that may be associated with both health care setting and mortality. While a portion of the effects seen may be related to patient-level differences in insurance selection or to any insurance, the results are unlikely solely explained by confounding. Among the uninsured, the Institute of Medicine^31^ estimated that 25% of mortality differences might be related to insurance status; however, even if correct, in the full population this would only account for a small proportion of the differences seen. In the current study, for example, differences were found between KP and the all-California population (uninsured population: 5.1%). In addition, the United States' uninsured population has also decreased substantially.^31-35^ The rollout of the Affordable Care Act was associated with a 52% decrease in uninsured rates among nonmembers in regions with KP, from 20.0% to 9.6% between 2010 and 2019; however, this was not accompanied by attenuation of mortality rates, either in the United States overall or differences between KP members vs nonmembers (KP: 20.5% lower in 2010 vs 21.5% in 2019).^36^ It would also seem unlikely to be explained by differences between the study population and its source regional population, given that the study population has a diverse payor mix (public [Medicare], commercial, and safety-net [Medicaid] systems) and closely approximates the region's underlying population by age, race, ethnicity, sex, educational achievement, household income, and a social vulnerability index.^5^

For potential causality, the findings address 8 of the 9 Hill criteria for likely causality,^37,38^ including the following: strength of the associations; consistency, across different relevant comparators, times, and diseases; specificity, for preventable diseases and effect size (larger for diseases with highly effective interventions and smaller for those with less-effective interventions); temporality, including disease-specific publications demonstrating relationships to multiple related population-health interventions^6-12^; biologic gradient, with the effects greatest for ages most exposed to disease risk interventions and for diseases with the most effective interventions, such as cardiovascular disease; plausibility, given that interventions address disease mechanisms and are supported by disease-specific randomized trials; coherence, across existing disease-specific studies, randomized trials, and within-population studies; and analogy. The only criterion not met is experimentation. Relating to plausibility, intervention effectiveness is related to uptake. For HEDIS (Healthcare Effectiveness Data and Information Set)-measured interventions among the 5 leading causes of death, high uptake is supported by the study setting being in the 90th percentile or greater for 57% of measures in 2010 and 49% of measures in 2019 vs 10% and 9% of measures for other reported commercial plans, respectively, with comparable data for Medicare plans.^25^ With regard to plausibility of effect estimate magnitudes, the life expectancy of 82.3 years is consistent with the average of 82.5 years across comparator nations for 2023. The study setting provided substantial opportunities for both exposure to population care and outcome ascertainment as more than 90% of KPNC members evaluated aged 65 years and older and more than 81% of people ages 40–64 years were members for at least 5 years (2023); the largest life expectancy contributions accrued at these ages, consistent with population health-prevention and treatment interventions. The mortality differences were largest for conditions with multiple modification strategies (eg, cardiovascular), smaller for those with only some effective prevention or treatment strategies (eg, across cancer types), and absent for less-modifiable causes of death (eg, dementia).^39-41^

Second, although life expectancy estimates are often interpreted as longevity predictions, they only summarize age-specific mortality in the recent past. While less useful for long-term forecasting, they are potentially informative here, as they reflect within-year health system practices and age-related effects. Third, death record linkages with US vital statistics may miss small percentages of deaths, estimated at less than 2%. Fourth, cause-of-death coding may vary across settings, although the sources used common national standards and similar trends were found for within-state comparisons. Fifth, life expectancy methods may underestimate preventive effects if recently implemented practices mainly modify future mortality. For example, new effective prevention or treatment strategies may not demonstrate commensurate mortality improvements for several years.

The population health concepts described are feasible and generalizable to other settings; they require mainly existing evidence-based recommendations, modern electronic medical records, and an organizational commitment to population outreach with follow-up. These processes are similar to every-6-month dental care reminders and follow-up, which are widely integrated into group, integrated, university, and individual practice settings. Although, to our knowledge, no data exist for direct mortality comparisons across different care funding models, generalizability is suggested by noting that 2 of the 4 top-ranked health plans in the National Committee for Quality Assurance use different care models from the study setting, with value-based care provider incentives, rather than directly employed clinicians.^42^ Other public funded models, such as the US Department of Veterans Affairs, also use center-specific strategies across over 100 medical centers, with extremely high rates for cancer screening, tobacco discontinuation, eye examinations, and other metrics, with minimal differences by patient demographics.^43^ Use of these mechanisms within these different systems also supports feasibility, given that they include people across multiple settings, states, communities, and centers. The ongoing integration of electronic medical records also increases the potential for generalizability. Electronic health record use between 2008 and 2021 increased from less than 9% to 96% for hospitals and from 17% to 78% for office-based physicians.^40^

In summary, a large, multicenter setting using multiple, high-uptake, evidence-based disease-management programs had markedly lower mortality rates from leading chronic diseases and avoidable causes of death, substantially longer projected life expectancy, and smaller demographic disparities than comparison populations.^39^ The causality criteria and consistency across comparators suggest that the associations are likely causal, rather than being explained largely by patient demographics, insured status, socioeconomic factors, or other confounders. These findings suggest that coordinated efforts using existing evidence and readily available technology represent a feasible approach for increasing health care value and decreasing avoidable deaths from chronic diseases within the United States.

## Supplementary Material

qxag184_Supplementary_Data

## Data Availability

For data sharing, the authors can provide relevant summary data used for the primary analyses in this article.
