# Supplementary material for: Associations of multiple evidence-based care strategies with disease mortality, life expectancy, and disparities in the United States
Source: Health Aff Sch. 2026 Jul 28;4(8):qxag184. doi: 10.1093/haschl/qxag184 (PMC13439128; doi:10.1093/haschl/qxag184)
Supplement: Supplementary file 1 [file qxag184_supplementary_data.zip › Health Affairs Supplement 2025_12_01.docx]

SUPPLEMENTARY FIGURES AND TABLES

Supplementary Table 1. Population Management Evaluations and Ratings (National Committee for Quality Assurance, 2019-2020), Kaiser Permanente, Northern California. (5 is highest rating)

| **Plan Details** | **Plan Details** | | |  | | | | | |  | **Score** |
| --- | --- | --- | --- | --- | --- | --- | --- | --- | --- | --- | --- |
|  |  | Prevention |  |  |  |  |  |  |  |  | 5.0 |
|  | Children and adolescent well-care | | | | | | | | | | 5.0 |
|  | Childhood immunizations status- combination 10 Did children receive all recommended immunizations by age 2? | | | | | | | | | | 5.0 |
|  | Adolescent immunizations: Combo 2 Did children receive all 3 recommended immunizations by age 13? | | | | | | | | | | 5.0 |
|  | BMI percentile assessment Did members ages 3 to 17 have their BMI assessed? | | | | | | | | | | 5.0 |
|  | Women’s reproductive health | | | | | | | | | | 5.0 |
|  | Prenatal checkups Did pregnant women have a prenatal visit in their first trimester or shortly after enrolling in a health insurance plan? | | | | | | | | | | 5.0 |
|  | Postpartum care Did women who gave birth have a postpartum visit three to eight weeks after delivery? | | | | | | | | | | 5.0 |
|  | Cancer screening | | | | | | | | | | 5.0 |
|  | Breast cancer screening Did women ages 50 to 74 get mammograms every two years? | | | | | | | | | | 5.0 |
|  | Colorectal cancer screening Were members ages 50 to 75 screened for colorectal cancer? | | | | | | | | | | 5.0 |
|  | Cervical cancer screening Did women ages 21 to 64 receive cervical cancer screening? | | | | | | | | | | 5.0 |
|  | Other preventive services | | | | | | | | | |  |
|  | Adult BMI assessment Did adult members ages 18 to 74 have their BMI assessed? | | | | | | | | | | 5.0 |
|  | Chlamydia screening Did sexually active women ages 16 to 24 et tested for chlamydia? | | | | | | | | | | 5.0 |
|  | Flu shots for adults Were members ages 18 to 64 vaccinated against the flu? | | | | | | | | | | 3.0 |
|  |  | Treatment |  |  |  |  |  |  |  |  | 4.5 |
|  | Asthma | | | | | | | | | | 3.0 |
|  | Asthma control Did people, ages 5 to 64, with persistent asthma have an appropriate ratio of asthma medications to help control their symptoms? | | | | | | | | | | 5.0 |
|  | Asthma drug management** Did people, ages 5 to 64, with persistent asthma take medications to control their asthma as prescribed? | | | | | | | | | | 1.0 |
|  | Diabetes | | | | | | | | | | 5.0 |
|  | Blood pressure control (140/90) Did diabetic members ages 18 to 75 have their blood pressure below 140/90 at their last visit? | | | | | | | | | | 5.0 |
|  | Eye exams Did diabetic members ages 18 to 75 have a retinal or dilated eye exam? | | | | | | | | | | 5.0 |
|  | Glucose control Did diabetic members ages 18 to 75 maintain their blood sugar level below 8 percent? | | | | | | | | | | 5.0 |
|  | Patients with diabetes – received statin therapy Did members ages 40 to 75 with diabetes who do not have cardiovascular disease receive a statin medication? | | | | | | | | | | 5.0 |
|  | Patients with diabetes – statin adherence 80% Did members ages 40 to 75 with diabetes who do not have cardiovascular disease stay on statin therapy as prescribed? | | | | | | | | | | 4.0 |
|  | Heart disease | | | | | | | | | | 4.5 |
|  | Patients with cardiovascular disease – received statin therapy Did males 21 to 75 and females 40 to 75 with cardiovascular disease receive a high or moderate-intensity statin medication? | | | | | | | | | | 5.0 |
|  | Patients with cardiovascular disease – statin adherence 80% Did males 21 to 75 and females 40 to 75 with cardiovascular disease stay on high or moderate-intensity statin therapy as prescribed? | | | | | | | | | | 3.0 |
|  | Controlling high blood pressure Did hypertensive patients ages 18 to 85 have their blood pressure controlled (i.e., for patients 18 to 59 a BP <140/90 mm Hg, for patients 60 to 85 with a diagnosis of diabetes a BP <140/90 mm Hg or a BP <150/90 mm Hg without a diagnosis of diabetes)? | | | | | | | | | | 5.0 |
|  | Mental and behavioral health | | | | | | | | | | 4.5 |
|  | Continued follow-up after ADHD diagnosis Did children ages 6 to 12 who were on ADHD medication for at least 210 days have at least two follow-up visits within 9 months? | | | | | | | | | | 4.0 |
|  | Depression: Adhering to medication for 6 months Did adult members with a new episode of depression take a prescribed antidepressant drug for at least 6 months? | | | | | | | | | | 3.0 |
|  | Follow-up after hospitalization for mental illness Were members hospitalized with a mental illness aged six and older followed up within a week after discharge? | | | | | | | | | | 5.0 |
|  | Follow-up after ED for mental illness Were members ages six and older with an ED visit for mental illness followed up within a week of their visit? | | | | | | | | | | 3.0 |
|  | Follow-up after ED for alcohol and other drug abuse or dependence Were members ages 13 and older with an ED visits for alcohol or other drug abuse or dependence followed up within a week of their visit? | | | | | | | | | | 5.0 |
|  | Alcohol or drug abuse or dependence treatment engaged Did alcohol- or drug-dependent members 13 and older receive two or more inpatient or outpatient care services within 34 days? | | | | | | | | | | 5.0 |
|  | Cholesterol and blood sugar testing for youth on antipsychotic medications Did members ages 1 to 17 who were on two or more antipsychotic medications have their blood sugar and cholesterol tested? | | | | | | | | | | 5.0 |
|  | First-line psychosocial care for youth on antipsychotic medications Did members ages 1 to 17 who had a new prescription for an antipsychotic medication have documentation of psychosocial care as first-line treatment? | | | | | | | | | | 4.0 |
|  | Other treatment measures | | | | | | | | | |  |
|  | Acute hospital utilization For members ages 18 and older, how many unplanned acute inpatient and observation stays occurred during the year? | | | | | | | | | | 5.0 |
|  | Observed-to-expected hospital readmissions For members ages 18-64, how many hospital stays were followed by an unplanned hospital readmission within 30 days after discharge? | | | | | | | | | | 3.0 |
|  | Emergency department utilization For members ages 18 and older, how many emergency department visits were unplanned during the year? | | | | | | | | | | 5.0 |
|  | Appropriate antibiotic use, adults with acute bronchitis Were adults ages 18 to 64 with acute bronchitis not prescribed antibiotics in the 3 days after their diagnosis, as recommended? | | | | | | | | | | 5.0 |
|  | Appropriate testing and care, children with sore throat Were children ages 3 to 18 with a sore throat given strep tests and appropriately prescribed antibiotics? | | | | | | | | | | 4.0 |
|  | Appropriate use of imaging studies for low back pain Were imaging tests appropriately not recommended for patients with a new episode of low back pain? | | | | | | | | | | 5.0 |
|  | Avoiding opioids at high dosage* Were members ages 18 and older not prescribed opioids at a high dosage for 15 or more days? | | | | | | | | | | 4.0 |
|  | Avoiding opioids from multiple prescribers and multiple pharmacies* Were members ages 18 and older who received prescription opioids for 15 or more days not prescribed opioids from four or more different prescribers and four or more different pharmacies? | | | | | | | | | | 2.0 |
|  | Steroid after hospitalization for acute COPD Did members 40 and older who were hospitalized or had an ED visit for chronic obstructive pulmonary disease receive systemic corticosteroids within 14 days of discharge? | | | | | | | | | | 5.0 |
|  | Bronchodilator after hospitalization for acute COPD Did members 40 and older who were hospitalized or had an ED visit for chronic obstructive pulmonary disease receive a bronchodilator within 30 days of their discharge? | | | | | | | | | | 5.0 |
|  | Appropriate antibiotic use, children with colds Were children ages 3 months to 18 years with colds not prescribed antibiotics up to three days after their diagnosis, as recommended? | | | | | | | | | | 5.0 |
| NCQA Accreditation as of June 30, 2019. * = NCQA recommends exercising caution when comparing HEDIS 2019 health plan performance on Use of Opioids at High Dosage (UOD) and Use of Opioids from Multiple Providers (UOP) due to health plan variation in denominator size and different state requirements.  **Some values include challenges with data availability | | | | | | | | | | | |
